# Molecular pedomorphism underlies craniofacial skeletal evolution in Antarctic notothenioid fishes

**DOI:** 10.1186/1471-2148-10-4

**Published:** 2010-01-06

**Authors:** R Craig Albertson, Yi-Lin Yan, Tom A Titus, Eva Pisano, Marino Vacchi, Pamela C Yelick, H William Detrich, John H Postlethwait

**Affiliations:** 1 Department of Biology, Syracuse University, 130 College Place, Syracuse, NY, 13244, USA; 2 Institute of Neuroscience, 1254 University of Oregon, Eugene, OR, 97403-1254, USA; 3 Dipartimento di Biologia, Università di Genova, Viale Benedetto XV, 5, 16132 Genova, Italy; 4 ICRAM, c/o Museo Nazionale dell'Antartide (MNA), Università di Genova, Viale Benedetto XV, 5, 16132 Genova, Italy; 5 Department of Oral and Maxillofacial Pathology, Tufts University, 136 Harrison Avenue, Boston, MA, 02111, USA; 6 Department of Biology, 134 Mugar Hall, Northeastern University, Boston, MA, 02115, USA

## Abstract

**Background:**

Pedomorphism is the retention of ancestrally juvenile traits by adults in a descendant taxon. Despite its importance for evolutionary change, there are few examples of a molecular basis for this phenomenon. Notothenioids represent one of the best described species flocks among marine fishes, but their diversity is currently threatened by the rapidly changing Antarctic climate. Notothenioid evolutionary history is characterized by parallel radiations from a benthic ancestor to pelagic predators, which was accompanied by the appearance of several pedomorphic traits, including the reduction of skeletal mineralization that resulted in increased buoyancy.

**Results:**

We compared craniofacial skeletal development in two pelagic notothenioids, *Chaenocephalus aceratus *and *Pleuragramma antarcticum*, to that in a benthic species, *Notothenia coriiceps*, and two outgroups, the threespine stickleback and the zebrafish. Relative to these other species, pelagic notothenioids exhibited a delay in pharyngeal bone development, which was associated with discrete heterochronic shifts in skeletal gene expression that were consistent with persistence of the chondrogenic program and a delay in the osteogenic program during larval development. Morphological analysis also revealed a bias toward the development of anterior and ventral elements of the notothenioid pharyngeal skeleton relative to dorsal and posterior elements.

**Conclusions:**

Our data support the hypothesis that early shifts in the relative timing of craniofacial skeletal gene expression may have had a significant impact on the adaptive radiation of Antarctic notothenioids into pelagic habitats.

## Background

Antarctic notothenioids are endemic to the Southern Ocean and probably evolved *in situ *from a sluggish, benthic perciform species beginning 40-60 Mya in the then temperate waters of the Antarctic continental shelf [1]. With the opening of the Drake Passage (~34-30 Mya), the establishment of the Antarctic Circumpolar Current, and a sharp drop in atmospheric carbon dioxide levels, the Southern Ocean became thermally isolated, began to cool, and attained its present frigid temperatures (-2 to +2°C) by the mid-Miocene (14-10 Mya) [1-8]. The rich, shallow-water, temperate fish fauna characteristic of the late Eocene (38 Mya) became largely extinct due to habitat destruction and changes in trophic structure, thus freeing ecological niches into which the notothenioids radiated [4].

The hallmark of the Antarctic notothenioid radiation is the evolution of secondary pelagicism, which is prominent in the family Nototheniidae [1] and common in the family Channichthyidae [9,10]. About 50% of notothenioid species now either live in or actively exploit pelagic habitats [11]. A significant obstacle facing notothenioids as they radiated into pelagic habitats was the ancestral absence of a swim bladder, the gas-filled chamber that most teleosts use to maintain buoyancy. In pelagic notothenioids, natural selection favored compensatory changes in the musculoskeletal system to achieve neutral buoyancy, including the replacement of densely mineralized bone with cartilage and connective tissue, decreased bone mineralization, and the accumulation of lipid deposits in muscle and connective tissues.

Changes in habitat were also accompanied by the evolution of distinct oral jaw morphologies and body shapes that accommodate shifts in diet and foraging behavior [12,13]. Trophic evolution among notothenioids resulted in two distinct pelagic ecotypes: true pelagics and benthopelagics [13]. True pelagics spend most of their time in the water column where they feed on microinvertebrates. They possess relatively short, protractile jaws for suction feeding and have few, large oral teeth arranged along a single tooth row (e.g., the Antarctic silverfish, *Pleuragramma antarcticum*). In contrast, benthopelagics spend much of their time on or close to the ocean floor but venture into the pelagic zone to forage on schools of small fish and macroinvertebrates. Most benthopelagic notothenioids have non-protractile, elongate jaws, a wide gape, and many, small oral teeth (e.g., the blackfin icefish, *Chaenocephalus aceratus*). This morphology enables benthopelagics to feed efficiently on krill and schools of small fishes by expanding their buccal cavity, and overtaking and sifting large mouthfuls of prey (e.g., ram feeding). Extant benthic notothenioids preserve the ancestral condition, exhibiting robust skeletons and heavily fortified jaws bearing several rows of large teeth (e.g., the yellowbelly rockcod,*Notothenia coriiceps*).

Notothenioids achieved secondary pelagicism by pedomorphism, the retention of ancestrally juvenile traits by adults in a descendant taxon [11]. Pedomorphism arises from heterochronic processes that alter the schedule of developmental events [14]. Striking examples of pedomorphic characters in pelagic and benthopelagic notothenioids include delayed and reduced skeletal ossification, retention of the notochord, reduced numbers of teeth and tooth rows, and reduction of the pterygoid process of the palatoquadrate [13,15-17].

As a first step toward understanding the molecular mechanisms that underlie skeletal reduction and morphological change in this group, we characterized early craniofacial development in a true pelagic species, *P. antarcticum *(family Nototheniidae), and a benthopelagic species, *C. aceratus *(family Channichthyidae). We describe shared derived aspects of craniofacial development in pelagic notothenioids that differ significantly from those observed in a benthic notothenioid species, *N. coriiceps *(family Nototheniidae), another percomorph species, *Gasterosteus aculeatus *(threespine stickleback, family Gasterosteidae), and a more distantly related outgroup, *Danio rerio *(zebrafish, family Cyprinidae). We found that pelagic notothenioid larvae exhibit a delay in osteogenic development, and that this heterochronic shift is associated with altered gene expression, including delayed expression of the osteogenic markers, *col1a1 and col10a1*, and prolonged expression of the cartilage differentiation gene, *col2a1*. These data provide a discrete molecular inroad into the mechanisms that underlie the evolution of this pedomorphic character. We also show that other changes in the patterning of the notothenioid trophic apparatus are due to apparent shifts in the relative timing of development of particular skeletal elements. These data are consistent with the hypothesis that heterochronic shifts in early craniofacial skeletal development have played significant roles in the adaptive diversification of Antarctic notothenioids.

## Results and Discussion

### Development of skeletal mineralization is delayed in Antarctic fish

**Figure 1:**
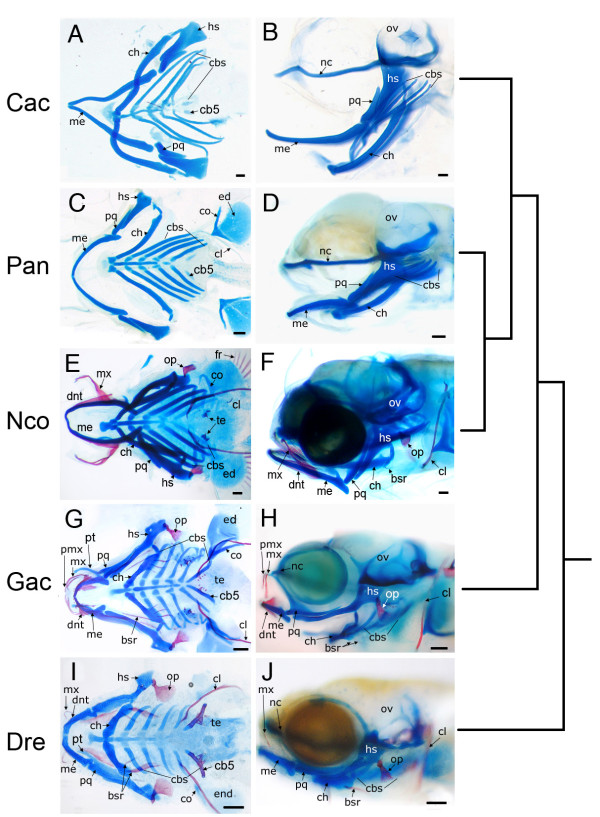
**Fish Specimens**. Cleared and stained skeletal preparations of the Antarctic silverfish (*P. antarcticum*), the blackfin icefish (*C. aceratus*), the yellowbelly rockcod (*N. coriiceps*), the threespine stickleback (*G. aculeatus*), and the zebrafish (*D. rerio*) at comparable stages of development. Bone is stained red with Alizarin red and cartilage is stained blue with Alcian blue. (A, B) *C. aceratus*, mid-larvae; (C, D)*P. antarcticum*, mid-larvae; (E, F) *N*. coriiceps, mid-larvae; (G, H) G. *aculeatus*, 10 dpf; (I, J) *D. rerio*, 6 dpf. Both ventral flat mounted (A, C, E, G, I) and lateral (B, D, F, H, J) views are shown. Note the lack of bony elements in pelagic notothenioid species compared to the benthic notothenioid and outgroups. The tree to the right of the images indicates evolutionary relationships. Abbreviations: bsr, branchiostegal rays; cbs, ceratobranchal cartilages; cb5, 5^th ^ceratobrachial cartilage; ch, ceratohyal; cl, cleithrum; co, corocoid; dnt, dentary; ed, endochondral disk; fr, fin rays; hs, hyosymplectic; me, Meckel's cartilage; mx, maxilla; nc, neurocranium; op, opercle; ov, otic vesicle; pmx, premaxilla; pq, palatoquadrate; pt, pterygoid process of the palatoquadrate; te, teeth. Scale bars, 100 μm.

Skeletal preparations were made of pelagic notothenioid larvae beginning at developmental stages just before hatching and continuing until just before yolk absorption and the onset of exogenous feeding (see Additional file 1), and compared to those of the benthic notothenioid and outgroup species. The results showed that pelagic notothenioid pharyngeal skeletons lack any mineralized tissues at developmental stages during which other species have begun to differentiate a well-formed bony skeleton (Fig. 1). Formation of mineralized tissue was not detected using Alizarin red staining, although developing cartilages had accumulated Alcian-staining cartilaginous material. We conclude that bone mineralization is delayed in developing pelagic, osteopenic Antarctic notothenioid embryos relative to outgroup fishes. To investigate the molecular genetic basis for this delayed ossification, we cloned and examined the developmental expression of genes involved in skeletal matrix formation.

### Nucleotide sequence similarity and phylogenetic reconstruction of collagen genes

**Figure 2:**
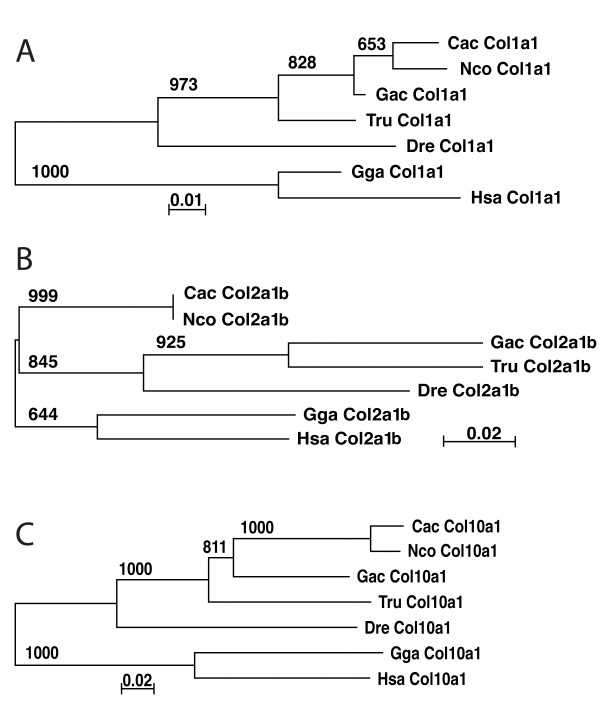
**Neighbor-joining tree for *col1a1 *(A), *col2a1b *(B) and *col10a1 *(C)**. Numbers on internodes are the frequency of each branch in 1,000 bootstrap replicates. Cac = *C. aceratus *(*col1a1*, FJ932592; *col2a1b*, FJ932595; *col10a1*, FJ932598), Nco = *N. coriiceps *(*col1a1*, FJ932593; *col2a1b*, FJ932596; *col10a1*, FJ932599), Gac = *G. aculeatus *(*col1a1*, FJ932594; *col2a1b*, FJ932597; *col10a1*, FJ932600), Tru = *Takifugu rubripes *(*col1a1*, N00041; *col2a1b*, N000077; *col10a1*, N000010), Dre = *Danio rerio *(*col1a1*, NP_954684; *col2a1b*, BC059180, *col10a1*, NP_001077296), Gga = *Gallus gallus *(*col1a1*, P02457; *col2a1b*, NP_989757; *col10a1*, P08125), Hsa = *Homo sapiens *(*col1a1*, NP_000079; *col2a1b*, NP_149162; *col10a1*, NP_000484).

To examine the expression of genes directing cartilage and bone formation, we cloned notothenioid derived collagen genes specific for the development of cartilage (*col2a1*) and bone (*col1a1 *and *col10a1*). The predicted amino acid sequence similarities among fishes were similar for all three collagen genes, with highest identities exhibited between the two notothenioids, the osteopenic *C. aceratus *and the strongly mineralized *N. coriiceps *(97%, 100%, and 95% for *col1a1*, *col2a1b*, and *col10a1*, respectively), whereas the lowest identities were generally found between fishes and tetrapod outgroups (chicken, human). The exception to this pattern was *col2a1b*, in which the *D. rerio *ortholog was the most highly divergent sequence for all comparisons, including tetrapods. The nucleotide sequences of *col2a1b *from *Chaenocephalus *and *Notothenia *differed at only two of 219 positions, a remarkably low rate of synonymous substitution in two lineages thought to have separated approximately 24 Mya [18]. Phylogenetic trees reflected accepted relationships among fish species, with *Danio *the most distantly related lineage to the *Takifugu *+ (*Gasterosteus *+ (*Chaenocephalus *+*Notothenia*) clade (Fig. 2A-C). *D. rerio col2a1b *exhibited an exceptionally long branch relative to other orthologs in this tree, reflecting accelerated amino acid sequence divergence in this lineage.

### Altered patterns of gene expression underlie reduced mineralization in pelagic notothenioid larvae

**Figure 3:**
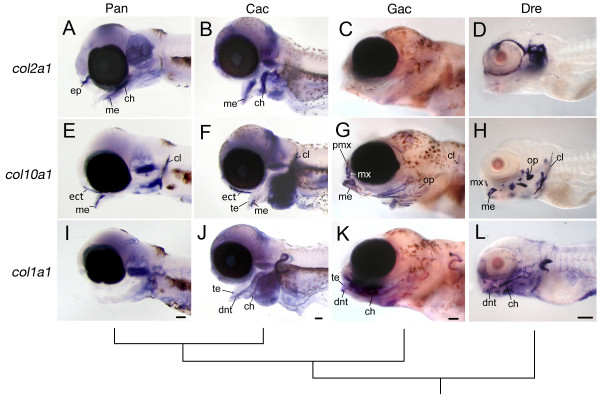
**Whole-mount *in situ *hybridization for *collagen *gene expression in the pharyngeal skeleton**. Lateral views are shown of embryos for *P. antarcticum*, *C. aceratus, G. aculeatus*, and *D. rerio *at comparable stages of development. (A, E, I) *P. antarcticum*, mid-larvae; (B, F, J) *C. aceratus*, mid-larvae; (C, G, K)*G. aculeatus*, 11 dpf; and (D, H, L) *D. rerio*, 5 dpf. Notothenioids retain strong *col2a1 *expression in the pharyngeal skeleton (A, B) well into larval development compared to both *G. aculeatus *(C) and *D. rerio *(D). Conversely, *col10a1 *(E, F) and *col1a1 *(I, J) expression is relatively weak or absent in the notothenioids. The tree represents the evolutionary relationship among species. Abbreviations: ch, ceratohyal; cl, cleithrum; dnt, dentary; ep, ethmoid plate; ect, ectopterygoid; me, Meckel's cartilage; mx, maxilla; op, opercle; pmx, premaxilla; te, teeth. Scale bars, 100 μm.

We observed prolonged developmental expression of the early cartilage marker *col2a1 *and reduced expression of the later developmental markers *col10a1 *and *col1a1 *in pelagic notothenioid larvae as compared to similarly staged stickleback and zebrafish. By 11 and 5 days post-fertilization (dpf), respectively, stickleback and zebrafish larvae exhibited very little *col2a1 *expression in the pharyngeal skeleton (Fig. 3C and 3D). In contrast, both pelagic notothenioid species exhibited strong expression of this cartilage marker through extended periods of larval development (Fig. 3A and 3B). The hypertrophic cartilage marker, *col10a1*, is normally expressed in terminally differentiated chondrocytes just before apoptosis and the onset of endochondral ossification [19]. This marker was discretely expressed throughout the pharyngeal skeleton in stickleback and zebrafish (Fig. 3G and 3H) but was limited to the cleithrum (cl) of the pectoral girdle, and to the upper and lower jaws (i.e., ectopterygoid, ept; Meckel's cartilage, me) in pelagic notothenioids (Fig. 3E and 3F). The osteoblast differentiation marker *col1a1 *was likewise ubiquitously expressed throughout the pharyngeal skeleton in both stickleback and zebrafish larvae (Fig. 3K and 3L). The same marker was only weakly expressed in the pharyngeal skeleton of the benthopelagic notothenioid, *C. aceratus*, and was virtually absent from the pelagic species, *P. antarcticum *(Fig. 3I and 3J).

**Figure 4:**
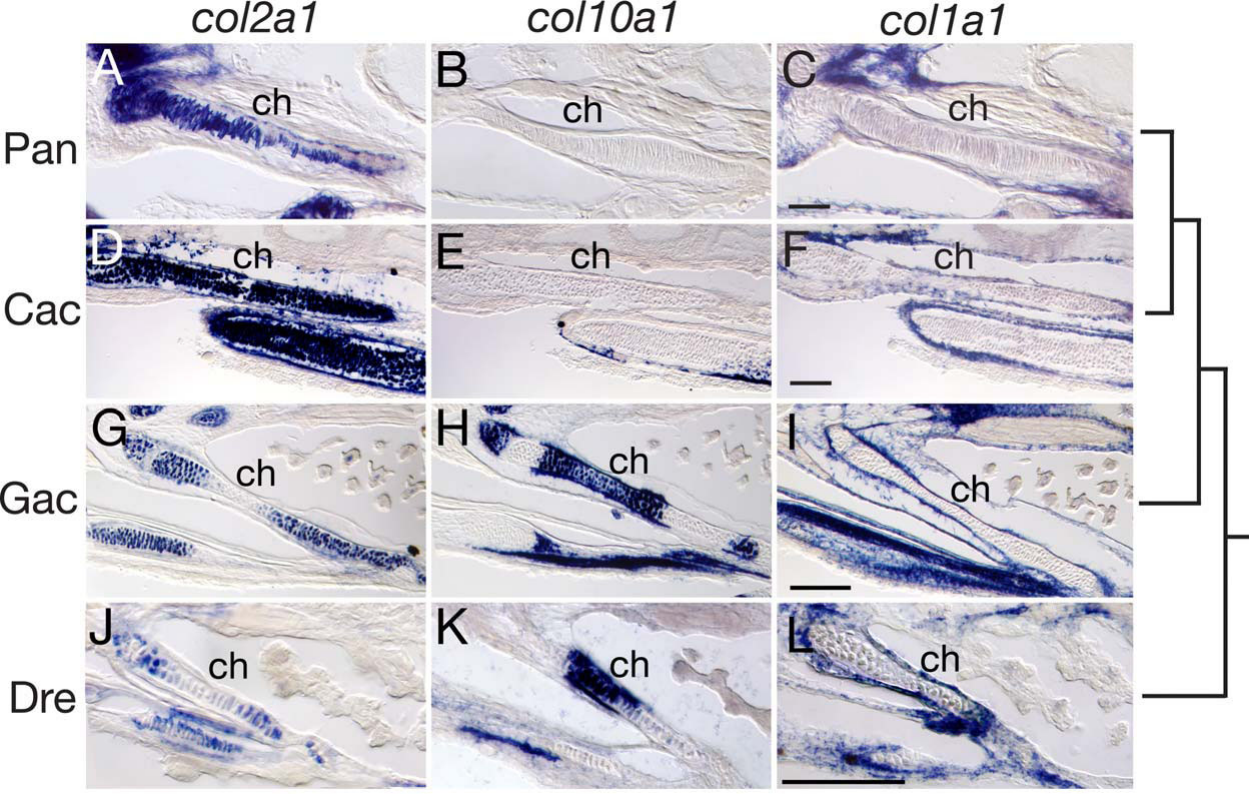
**Ceratohyal *collagen *gene expression in *P. antarcticum*, *C. aceratus*, *G. aculeatus*, and *D. rerio *detected by *in situ *hybridization on cryosections**. (A-C) *P. antarcticum*, mid-larvae; (D-F) *C. aceratus*, mid-larvae; (G-I) *G. aculeatus*, 11 dpf; (J-L) *D. rerio*, 6 dpf. Sections through the ceratohyal cartilage (ch) are shown in the ventral view. Notothenioid larvae show ubiquitous expression of *col2a1 *throughout the ch (A, D), whereas *col2a1 *expression is restricted to the distal ends of the cartilage in both *G. aculeatus *(G) and *D. rerio *(J). The *col10a1 *gene is expressed in hypertrophic chondrocytes in the ch in *G. aculeatus *(H) and *D. rerio *(K), whereas its expression is absent from the ch in *P. antarcticum *(B) and *C. aceratus *(E). The *col1a1 *gene is weakly expressed in the periosteum around the ch of both *P. antarcticum *(C) and *C. aceratus *(F) relative to the strong expression observed in *G. aculeatus *(I)and *D. rerio *(L). The tree represents the evolutionary relationship among species. Scale bars, 100 μm.

This pattern of delayed osteogenic gene expression was also evident in sectioned specimens (Fig. 4). Pelagic notothenioids exhibited strong *col2a1 *gene expression throughout the ceratohyal cartilage (ch, Fig. 4A and 4D), whereas in similarly staged stickleback and zebrafish *col2a1 *expression was restricted to the proximal and distal ends of this skeletal element (Fig. 4G and 4J). Expression of *col10a1 *was absent from the ceratohyal cartilage in notothenioid larvae (Fig. 4B and 4E), but was strongly expressed in the putative hypertrophic domain of the ceratohyal in stickleback and zebrafish (Fig. 4H and 4K). Likewise, strong expression of *col1a1 *was observed in the perichondrium surrounding the ceratohyal cartilage in both stickleback and zebrafish (Fig. 4I and 4L), while only weak perichondrial *col1a1 *expression was observed in notothenioid larvae (Fig. 4C and 4F). Although only single representative staged specimens are presented here, the observed trends were consistent over extended periods of larval development. In toto, these data are consistent with a delay in the osteogenic developmental program relative to the chondrogenic program in the two pelagic notothenioid species, relative to the other fishes examined.

**Figure 5:**
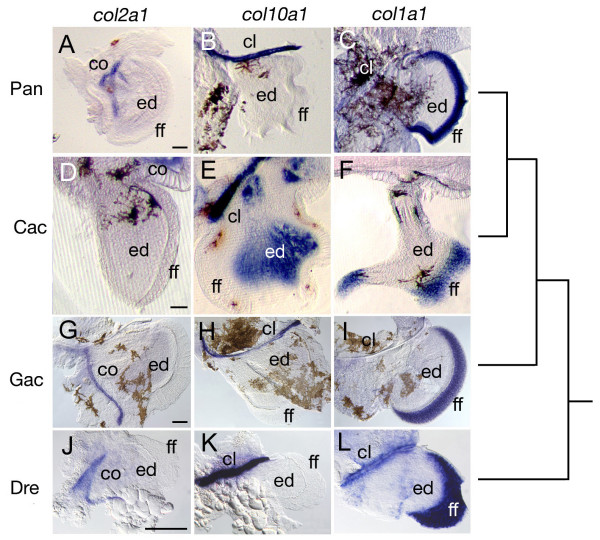
**Expression of *collagen *genes in the pectoral fins of *P. antarcticum*, *C. aceratus*, *G. aculeatus*, and *D. rerio *embryos, detected by whole-mount *in situ *hybridization**. (A-C) *P. antarcticum*, mid-larvae; (D-F) *C. aceratus*, mid-larvae; (G-I) *G. aculeatus*, 7 dpf; (J-L)*D. rerio*, 3 dpf. Images are of flat-mounted (A-C, G-L) or vibratome-sectioned (D-F) pectoral fins. Expression of *col2a1 *is observed in the scapulocoracoid (co) of all species (A, D, G, J). Likewise expression of *col10a1 *(B, E, H, K) and *col1a1 *(C, F, I, L) is observed in the cleithrum (cl), and *col1a1 *is expressed in the fin fold (ff) of all species. *C. aceratus *exhibits a unique pattern of *col10a1 *expression in the endochondral disk (ed) (E). Thus, gene expression during fin development appears to be largely conserved among these species. The tree represents the evolutionary relationship among species. Scale bars, 100 μm.

In contrast to the pharyngeal skeleton, the development of the appendicular skeleton was relatively conserved among fish species (Fig. 5). In all species examined, *col2a1 *expression was observed in the scapulocoracoid (co), *col10a1 *was expressed in the cleithrum (cl), and *col1a1 *expression was observed in the fin fold (ff). The only notable difference among species was strong expression of *col10a1 *in the endochondral disk (ed) of *C. aceratus *(Fig. 5E). We did not observe expression of *col10a1 *in this tissue in the other species at any stage, and expression of this marker appeared in the fins of *C. aceratus *only at later stages of larval development. The implications of potential novel gene expression in the fins of certain notothenioid species are interesting given the importance of fins in the adaptive radiation of this group [12], but the anatomical consequences of this expression pattern are not yet known.

These data suggest a tradeoff between the chondrogenic and osteogenic programs during craniofacial (but not appendicular) skeletal development. Skeletal development is highly conserved among vertebrates, and pathways leading to either cartilage or bone formation are tightly linked. Condensing cartilage progenitor cells express *sox9*, which regulates *col2a1 *expression [20,21] and is necessary and sufficient for cartilage differentiation [22-24]. Osteoprogenitor cells express *runx2*, which modulates the expression of bone matrix genes including *col1a1 *and is necessary and sufficient for bone formation [23,25]. Both *sox9 *and *runx2 *can be expressed in the same cells, and it is the relative amount of each that specifies cell fate [23]. Runx2 binds to *col10a1 *and *ihh *promoters [25] to activate their expression in terminally differentiated hypertrophic chondrocytes [19,26]. Secreted *ihh *stimulates surrounding perichondrial osteoblasts to secrete Pthrp [27], which diffuses back to prehypertrophic chondrocytes, binds to its receptor Pth1r, and blocks further chondrocyte maturation. Thus, pedomorphic bone development and changes in *collagen *gene expression patterns in pelagic notothenioid species could be due to one or more mutations affecting these regulatory genes.

In addition to early pattering mechanisms, evolved bone loss in notothenioids might involve later developmental events. Bone development continues throughout the life of an individual, through balanced processes of bone growth and bone remodeling. Fish regulate skeletal growth and remodeling in much the same way as other vertebrates, through coordinated activities of bone forming cells (osteoblasts) and bone resorbing cells (osteoclasts) [28]. It is possible that decreased bone density in notothenioids, which begins early in life, may be maintained later in development by modulating osteoblast and osteoclast activities. Because osteoporosis in humans results from an unfavorable balance between bone deposition by osteoblasts and bone resorption by osteoclasts, these considerations provide fertile ground for future investigations.

### Pedomorphosis in notothenioid skeletal patterning

Morphological analyses revealed several pedomorphic traits in notothenioid species relative to stickleback and zebrafish. The most conspicuous differences between notothenioid and outgroup pharyngeal skeletons were the absence of the pterygoid process of the palatoquadrate and the concomitant expansion of ventral cartilages. This was most pronounced in the benthopelagic species *C. aceratus*, where the anterior and ventral pharyngeal cartilage elements (Meckel's cartilage and ceratohyal) were well formed and elongated before any other element appeared (see Additional file 1). This developmental sequence is distinct from that observed in both stickleback and zebrafish, where the palatoquadrate (dorsal element) forms almost simultaneously with Meckel's cartilage (ventral element), followed soon after by the ceratohyal [29]. The pterygoid process of the palatoquadrate forms relatively late in zebrafish and stickleback, after most of the other pharyngeal cartilages have begun to develop, but is well formed at the beginning of larval development. In notothenioids, the pterygoid process is conspicuously absent throughout extended periods of larval development, and in adults this structure (i.e., ectopterygoid) is greatly reduced in size [13].

Factors that pattern the pharyngeal skeleton along the D-V axis have been well studied in a variety of models, and include Endothelin (Edn), an important signaling molecule for D-V patterning of the zebrafish pharyngeal skeleton [30,31]. The *edn1 *gene is expressed in the ventral pharyngeal endoderm and is thought to signal through *hand2 *to promote the development of ventral arch structures (i.e., lower jaw), while inhibiting the expression of *bapx1 *and *eng2*, which are expressed in the dorsal pharyngeal pouch [30,32]. An enticing model of notothenioid jaw development would predict that over-expression of ventral factors (e.g., *edn1*) in the pharyngeal endoderm leads to the attenuation of dorsal signaling molecules, and the development of longer lower jaws and reduced upper jaw elements. Alternatively, the observed attenuation of the dorsal pterygoid process in notothenioids might be due to more localized signals, such as those regulating stomodeal development. In zebrafish, the pterygoid process develops from a subset of cranial neural crest cells that condense along the dorsal ectoderm of the stomodeum, the presumptive mouth opening [33]. Abrogation of Hedgehog signaling at the developing midline disrupts the specification of the stomodeum, and the condensation of cells fated to become the pterygoid, suggesting that the stomodeum may be a critical signaling center for pterygoid development.

Notothenioid larvae were also distinguished from outgroup species by the conspicuous absence of the most posterior pharyngeal cartilage, the fifth ceratobranchial (cb5). This bilaterally paired element forms the pharyngeal 'jaw' in both percomorph (e.g., notothenioids and stickleback) and cypriniform (e.g., zebrafish) fishes, bears teeth, and is used to processes prey gathered by the oral jaws. In most bony fishes, development of this cartilage is accelerated; cb5 forms at the same time as cb3 and before cb4, and is one of the first elements to be mineralized in the head [29]. In notothenioid adults, however, cb5 is significantly reduced [34], which we show here to be associated with a developmental delay in the formation of cb5. Notothenioid larvae possess only a rudimentary cb5 (Fig. 1), and it is the last pharyngeal cartilage to form, well after the development of more anterior cb cartilages. At the latest developmental stages examined, only a few pharyngeal teeth had formed in *C. aceratus *and *N. coriiceps *larvae, and no teeth had developed on the oral or pharyngeal jaws of *P. antarcticum *larvae, whereas comparably staged stickleback and zebrafish possessed well-mineralized cb5s bearing several teeth (Fig. 1). A-P patterning of the pharyngeal skeleton is determined by nested, overlapping expression of various *hox *genes [35-39], and it is possible that mis-expression of posterior *hox *genes might play a role in the delayed development of cb5 in notothenioid larvae. Alternatively, the primary mechanism leading to the reduction of cb5 could be due to signals emanating from tissues surrounding *hox*-positive cranial neural crest cells, including the pharyngeal endoderm or adjacent mesoderm [40-42]. Each of these hypotheses remains to be tested in a more thorough analysis of A-P patterning of the notothenioid pharyngeal skeleton.

Each notothenioid ecotype examined here exhibited relatively elongated lower jaws, absent pterygoid processes, and reduced cb5s as larvae (Fig. 1), suggesting that these traits were present in the common notothenioid ancestor and evolved independently from the bone mineralization phenotype. The pterygoid process of the palatoquadrate and the pharyngeal jaw play significant roles in the collecting and processing of pelagic prey [43-45]. The pterygoid process articulates with the maxilla and is predicted to play an important role during jaw protrusion [43]. The observation that the ectopterygoid is one of the few bones in the head where an early osteogenic program is retained in pelagic notothenioids (e.g., *col10a1 *expression, Fig. 2), likely reflects the importance for this bone during suction feeding. The pharyngeal jaw is used for both the mastication and laceration of prey in fishes [45]. While pharyngeal mastication is likely not important for suction feeding notothenioids [44], a reduced fifth ceratobranchial may have affected the efficiency to which the pharyngeal jaws are able process prey via laceration (e.g., the ability to break down the exoskeleton of pelagic crustaceans [45]). Whether a delay in the development of either of these structures represents a developmental constraint that has shaped patterns of diversification as nototheniods radiated to fill pelagic foraging niches remains an open question.

## Conclusions

**Figure 6:**
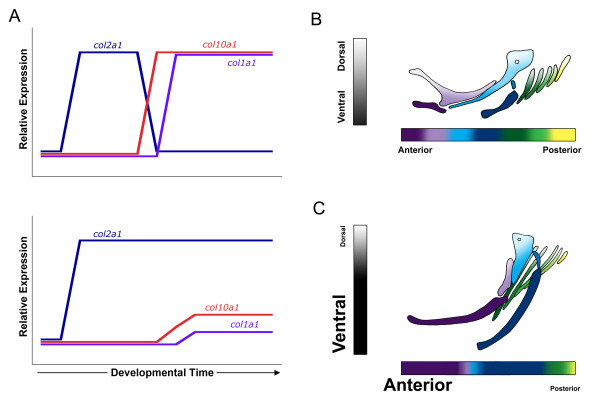
**Schematic illustration of notothenioid craniofacial evolution via heterochrony**. Panel A summarizes results for the relative patterns of each *collagen *gene expression during notothenioid larval skeletogenesis. Expression in *G. aculeatus *and *D. rerio *follows a typical vertebrate pattern, with *col2a1 *expressed first in differentiating chondrocytes, followed by a down regulation of *col2a1 *and subsequent up regulation of the bone markers, *col10a1 *and *col1a1 *(A, top panel). The notothenioid pattern is quite different, with sustained high levels of *col2a1 *expression throughout later periods of larval development, expression of *col10a1 *limited to a small subset of elements, and weak *col1a1 *expression throughout the pharyngeal skeleton (A, bottom panel). The x-axis denotes developmental time starting just before chondrocyte differentiation, and the y-axis represents relative (not quantitative) levels of *collagen *expression. Panels B and C depict a model for anterior-posterior and dorsal-ventral patterning of the pharyngeal skeleton. Compared to stickleback and zebrafish, the developmental program in notothenioid species is shifted toward the ventral components (darkly shaded) of the anterior-most craniofacial elements (purple and blue). This shift results in the development of rostral-caudally expanded Meckel's (purple) and ceratohyal (blue) cartilages, and reduced posterior cartilage elements (i.e., fifth ceratobranchial cartilage, yellow).

Notothenioids dominate the near-shore habitat of Antarctica and are an important example of an adaptive radiation in marine fishes [1,5,46]. While the ecological and environmental shifts that have precipitated these events are well understood, virtually nothing is known about the molecular and developmental changes that underlie adaptive variation in notothenioid feeding architecture. We propose that salient aspects of the evolution of the notothenioid craniofacial skeleton can be explained by discrete shifts in early developmental genetic scheduling (Fig. 6).

A small but growing number of studies have described heterochrony at the molecular level. The evolution and development of the vertebrate limb, for example, provides several examples of developmental genetic heterochrony. Shifts in the relative timing of expression of *Shh *in the zone of polarizing activity are correlated with decreased digit number in lizards [47], while prolonged *Fgf8 *expression in the apical ectodermal ridge is correlated with the development of extra phalangeal elements in dolphins [48]. Excellent opportunities to study genetic heterochrony are also provided by naturally occurring wing color mutations in butterflies [49], and by induced mutations in *C. elegans *that affect developmental timing [50]. Genetic heterochrony may also explain differential expression of cone opsin genes in cichlid fishes, providing a potentially potent mechanism through which visual sensitivities can evolve in this group [51]. In spite of these putative exemplars the number of described molecular mechanisms that underlie evolutionary change by heterochrony are disproportionately small given the prominence of this process in discussions of morphological evolution [14]. Here we show that the evolution of bone loss in Antarctic notothenioids can be explained in part by the prolongation of the early chondrogenic developmental pathway through extended periods of larval development. These data offer a molecular inroad into the larger signaling cascades that modulate bone density, a trait that is linked to the adaptive radiation of Antarctic notothenioids [1,52]. In addition to the evolutionary implications, understanding these mechanisms could provide insights into the development of human osteopenia and suggest effective therapies to prevent and/or treat this disease [53].

## Methods

### Collection and maintenance of fish species

A clutch of benthopelagic icefish embryos [keyed as *Chaenocephalus aceratus *at ~3 months postfertilization [54,55]] was collected near Brabant Island, Antarctica, on June 27, 2001. Specimens were reared at -1.5°C in the aquarium at Palmer Station Antarctica and sampled daily for two months. *Pleuragramma antarcticum *embryos and larvae were collected beneath the sea ice in Terra Nova Bay, Antarctica, in November, 2005 [56]. *Notothenia coriiceps *embryos were produced by in vitro fertilization in June, 2008 and reared at Palmer Station and at the University of Oregon. Unfortunately, relatively few *N. coriiceps *embryos survived through larval stages, and insufficient numbers were available for *in situ *hybridization analyses. Comparative staging of notothenioid samples is described in Additional file 1. Staged embryos were also collected from an anadromous population of the closely related threespine stickleback [57] and the distantly related zebrafish. Both of these species were reared and bred at the University of Oregon as described [58]. Experimental research conducted on these animals was performed according to protocols approved by the Institutional Animal Care and Use Committees (IACUC) at Syracuse University (#07-024) and the University of Oregon (#07-25A).

### Skeletal terminology and preparations

Throughout this report we refer to the "craniofacial skeleton" and "pharyngeal bones", common anatomical terms that are widely accepted and recognizable across multiple disciplines. For clarity, it is worth pointing out that by the craniofacial skeleton we are referring to the entirety of the cranial skeleton (i.e., chondrocranium, sphlanchnocranium and dermatocranium) [59], and by pharyngeal bones we are referring to those bones derived from the embryonic pharyngeal arches, including the mandibular and hyoid arches as well as posterior gill arches. Cartilage and bone staining was performed using the two-color acid-free protocol [60]. Samples were micrographed with a Zeiss Axiocam digital imaging system mounted to an M2 Bio stereomicroscope (Zeiss). Image processing used Adobe Photoshop CS4.

### Cloning and phylogenetic reconstruction of notothenioid collagen genes

Primers for genomic DNA amplification were based on *Gasterosteus aculeatus *genomic assembly BROAD S1, ENSEMBL version 49 http://www.ensembl.org/Gasterosteus_aculeatus/index.html. Primers were GACcol1a1F1/R1 GTGGCGGATTTGACCTCGGATTCAT/CACCGCTCTTCCAGTCAGGGTTGC, GACcol2a1F1/R1 ATGTCGGCCTTCGCTGGTCTGG/ACTCGGGGTGGCACAGCTTCAGGT, and GACcol10a1F1/R1 CTAAGGGAAACAAGGGAGATCAGG/GAAGCAATGAGGAACCCAGAGAA. Amplicon lengths were 238 bp, 220 bp, and 876 bp for *col1a1, col2a1b*, and *col10a1*, respectively. Genomic DNA from *C. aceratus *and from *N. coriiceps *was used as the PCR template.

PCR products were separated on 1% agarose gels, excised, and centrifuged at 14,000 *g*. Cloning was performed using 2 μl of the resulting supernatant and the TOPO Cloning Kit for Sequencing (Invitrogen, Carlsbad, CA) following the manufacturer's protocol. The DNA inserts of clones were sequenced by automated methods, and nucleotide sequences were translated to amino acids using Editseq (DNASTAR, INC., Madison). Predicted amino acid sequences were aligned and 1,000 Neighbor-joining bootstrap replicates performed using CLUSTALX 2.0 [61]. The bootstrap consensus tree was viewed using Njplot [62].

### In situ hybridization analysis

Whole-mount *in situ *hybridization analyses were performed as previously described [60]. Sense and anti-sense riboprobes were made from notothenioid clones. Selected whole mount specimens were sectioned after *in situ *hybridization using a vibrating microtome (Vibratome 1500, The Vibratome Company, St. Louis, MO). After hybridization, embryos were transferred directly into gelatin/albumin embedding solution [2.2 g gelatin (TYPE B) in 450 ml PBS; 135 g chick egg albumin; 90 g sucrose] for several minutes before transfer into molds containing fresh embedding solution plus 2.5% glutaraldehyde fixative. The molds were covered with cellophane, and the samples allowed to harden overnight at 4°C. The following day blocks were cut, mounted to the sectioning dish with super glue, and sectioned at 40 μm.

*In situ *hybridization was also performed on cryostat-sectioned material. Fixed material was embedded in 1.5% agar and 5% sucrose and stored in 30% sucrose at 4°C overnight. Cryostat sections (16 μm) were cut, transferred to Fisherbrand Superfrost/Plus microscope slides (Pittsburgh, PA), and stored at -20°C. Subsequent steps followed closely the method for whole-mount *in situ *hybridization.

## Authors' contributions

RCA, PCY, HWD and JHP contributed to the conception of this study. RCA and JHP designed the experiments. RCA and Y-LY generated cleared and stained bone preparations of staged specimens, and performed *in situ *hybridization experiments. TAT cloned notothenioid *collagen *genes and carried out the phylogenetic reconstructions. EP and MV generated the developmental series of *P. antarcticum*. HWD generated the developmental series of *C. aceratus*. RCA wrote the manuscript. All authors read, revised and approved the final manuscript.

## Supplementary Material

Additional file 1 **Staging of craniofacial skeleton development in pelagic notothenioids (supp1.docx)**. Three stages of craniofacial development are illustrated for both pelagic notothenioid species. From these data comparable stages were identified in zebrafish. Click here for file
