# Supplementary material for: Molecular pedomorphism underlies craniofacial skeletal evolution in Antarctic notothenioid fishes
Source: BMC Evol Biol. 2010 Jan 6;10:4. doi: 10.1186/1471-2148-10-4 (PMC2824663; doi:10.1186/1471-2148-10-4)
Supplement: Additional file 1 — Staging of craniofacial skeleton development in pelagic notothenioids (supp1.docx). Three stages of craniofacial development are illustrated for both pelagic notothenioid species. From these data comparable stages were identified in zebrafish. [file 1471-2148-10-4-S1.DOCX]

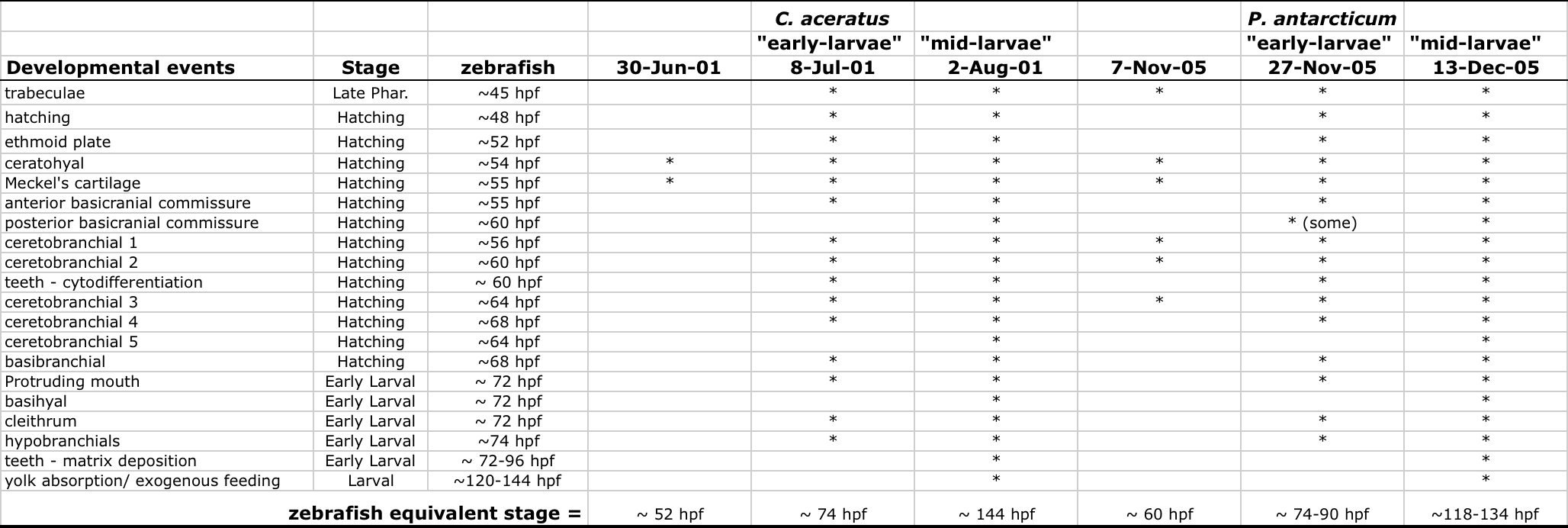


**Table S1.** Developmental staging as compared to zebrafish. Zebrafish staging was based on (19) and personal observations. Because of their unique evolutionary history and the environment in which they develop (e.g., cold and constant dark), notothenioids have evolved modifications to many structures and organ systems making comparisons to tropical zebrafish difficult. We were thus mainly limited to the developmental sequence and timing of pharyngeal cartilage development. Aside from slight sequence changes in the timing of development of the anterior (i.e., Meckel’s cartilage) and posterior (i.e., cb5) most elements, we found development of pharyngeal skeleton to be largely conserved compared to zebrafish and other teleost species (e.g., stickleback, cichlids, data not shown). We feel confident that our staging system provides a framework in which to make appropriate comparisons of craniofacial development in these species.

*C. aceratus* larvae were reared in aquaria and sampled periodically for ~40 days. Based on the presence and absence of structures we were able to stage *C. aceratus* samples collected on June 30 and July 08, 2001 as being equivalent to zebrafish stages ~52 hpf and ~74 hpf, respectively. This allowed us to apply a developmental ‘clock’ to other samples. *P. antarcticum* larva fixed on November 07 and 27, 2005, were collected beneath the sea ice in Terra Nova Bay, Antarctica, as described (63) and therefore showed a range of developmental stages. Nevertheless, we were able to confidently stage these samples to a relatively narrow range of equivalent zebrafish stages.


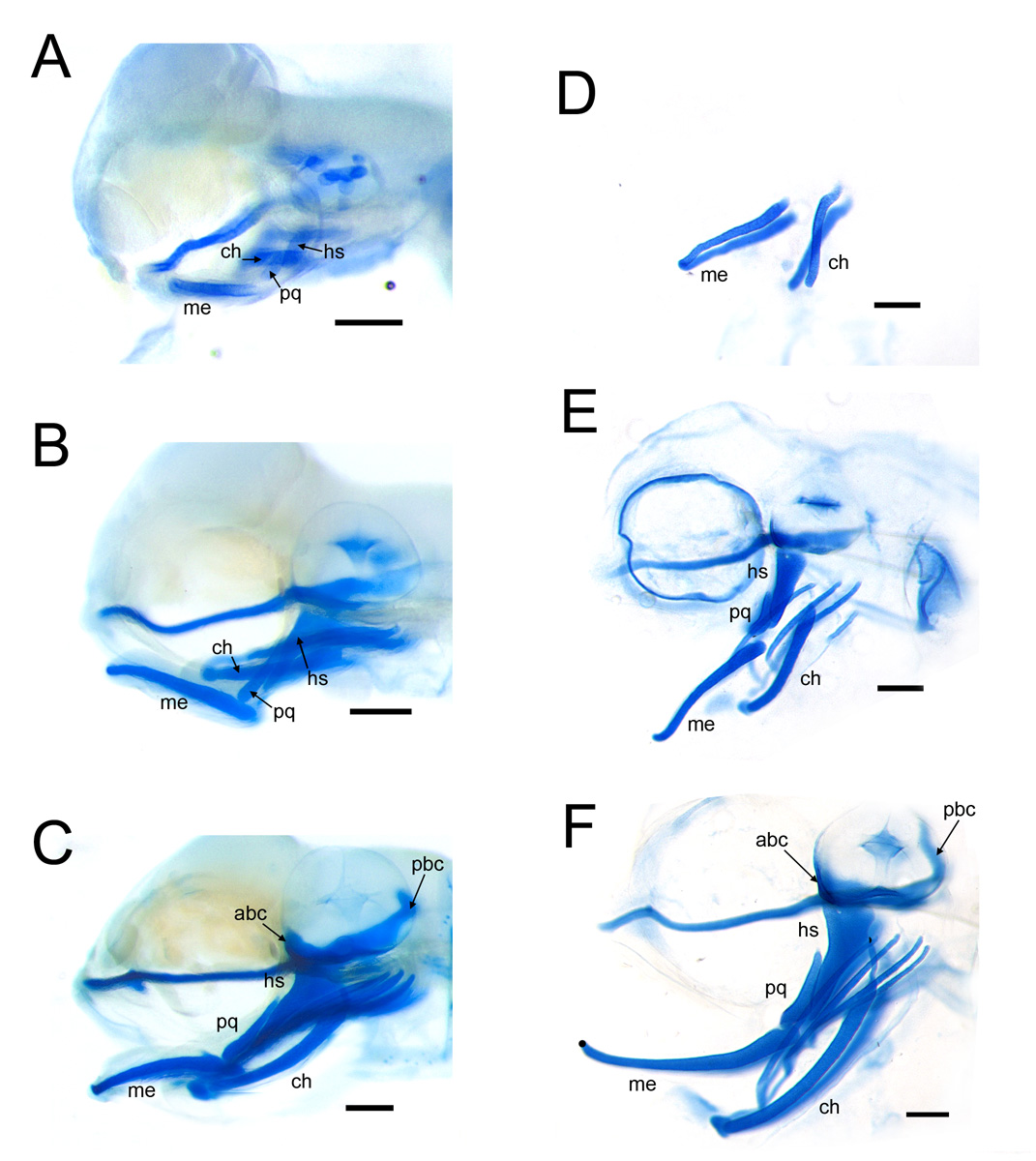


**Fig. S1.** Developmental stages of *P. antarcticum* (A-C) and *C. aceratus* (D-F). (A, C) Prehatching stages, equivalent to stages ~60 hpf and ~52 hpf, respectively, of the zebrafish hatching period (19). (B, E) Early-larvae stages, equivalent to ~74-90 hpf of the zebrafish larval period. Since *C. aceratus* specimens were systematically collected from a single clutch over the course of ~40 day, staging was a bit more straightforward. The *C. aceratus* specimen in (E) is 8 days older than that depicted in (D); it also corresponds to approximately zebrafish stage ~74 hpf. This is the protruding mouth stage in zebrafish and is characterized by the anterior extension of the jaw just past the eye. Based on these two time-points (i.e., D and E), we inferred that each day of development in notothenioids (at –1.5 ° C) corresponds to 2.5 hrs of development in zebrafish (at 28.5° C). (C, F) Mid-larvae stages, equivalent to zebrafish stage 5-6 dpf. The specimen in (F) is 24 days older that (E), and should therefore be at a similar stage as a 6 dpf zebrafish larvae. This is consistent with the presence of teeth in *C. aceratus* larvae at this stage. Staging of *P. antarcticum* was less straight forward, as samples represented natural populations of embryos collected beneath the sea ice (63). The sample in (A) was collected from Terra Nova Bay, Antarctica, on November 7, 2005, and was determined to be equivalent to zebrafish stage ~60 hpf. The sample in (B) was collected 20 days later, but there was clearly a range of developmental stages present in this collection. In particular, there were differences in the degree of mouth protrusion. This particular fish represented one of the younger samples, and based on the presence and absence of individual skeletal structures (Table S1), was determined to be at approximately the same stage as *C. aceratus* in panel (E). Fish collected on November 27 were subsequently reared for another 16 days in aquaria, and, based on the developmental “clock” applied to *C. aceratus*, these fish should be equivalent to zebrafish stage ~5 dpf. As before, this collection contained a range of stages, and the specimen depicted in panel (C) was one of the older individuals equivalent to zebrafish stage ~5.5 dpf.

Beyond specific developmental stages, it is important to note that the yolk sac was nearly absorbed in the oldest notothenioid specimens sampled, marking the onset of exogenous feeding. In both zebrafish and stickleback, and indeed most teleost species, the craniofacial skeleton is well mineralized by this developmental event to accommodate the functional demands of feeding. The observation that notothenioids lack this mineralization at hatching is significant and supports our conclusion that the osteogenic developmental program is delayed in these species. Abbreviations: abc, anterior basicranial commissure; ch, ceratohyal; hs, hyosymplectic; me, Meckel’s cartilage; pbc, posterior basicranial commissure; pq, palatoquadrate. Scale bars, 200 μm.
